# Spatial simulation of autologous cell defection for cancer treatment

**DOI:** 10.1093/emph/eoad042

**Published:** 2023-11-22

**Authors:** Jibeom Choi

**Affiliations:** Department of Applied Mathematics, College of Applied Sciences, Kyung Hee University, Yongin 17104, Republic of Korea

**Keywords:** cancer, evolution, autologous cell defection, evolutionary medicine

## Abstract

Cancer cells are highly cooperative in a nepotistic way and evolutionarily dynamic. Present cancer treatments often overlook these aspects, inducing the selection of resistant cancer cells and the corresponding relapse. As an alternative method of cancer elimination, autologous cell defection (ACD) was suggested by which modified cancer cells parasitically reliant on other cancer cells are implemented to the cancer cluster. Specifically, modified cancer cells should not produce costly growth factors that promote the growth of other cancer cells while receiving the benefit of exposure to such growth factors. Analytical models and rudimentary experiments up to date provide the medical feasibility of this method. In this study, I built comprehensive spatial simulation models by embracing the effects of the multiple growth factors, the Warburg effect, mutations and immunity. The simulation results based on planar spatial structures indicate that implementation of the defective modified tumours may replace the existing cancer cluster and defective cells would later collapse by themselves. Furthermore, I built a mathematical model that compares the fitness of the cells adjacent to the hypertumour–cancer interface. I also calculated whether anticancer drugs that reduce the effects of the growth factors promote or demote the utility of ACD under diverse fitness functions. The computational examination implies that anticancer drugs may impede the therapeutic effect of ACD when there is a strong concavity in the fitness function. The analysis results could work as a general guidance for effective ACD that may expand the paradigm of cancer treatment.

## INTRODUCTION

Cancer is detrimental to the body. However, with respect to the cancer cells themselves, they are highly cooperative [1, 2]. Cancer cells produce diffusible cancer growth factors (CGFs) such as nerve growth factor, epidermal growth factor or platelet-derived growth factor that promote the growth of the conspecifics and suppress the normal cells (alien to the cancer cells) [2–4]. Until mutated cells attain the full hallmarks of cancer [5], partially mutated cells may cooperate for their progression [1]. For example, type A cancer cells may produce growth factor 1, while the adjacent type B cancer cells produce growth factor 2 where both growth factors are beneficial for cancer growth. In this respect, cancer is ambivalent (defective to the body, cooperative among themselves).

If cancer is an outcome of cooperation among the conspecifics, then there could be defectors of the cooperation that jeopardize the fitness of the cancer clusters [6]. For example, some cancer cells may benefit from receiving growth factors while not producing them. As the production of growth factors entails the cost, those cancer cells that do not produce CGFs but take advantage of the other CGF-producing cancer cells can be considered as the tumours of the tumours [2]. These parasitic cells of the tumour cluster were conceptualized as hypertumour [6]. Hypertumour was speculated to be one of the reasons of Peto’s paradox that there is no significant correlation between body size (or longevity) and cancer prevalence across species [6–8]. If larger animals can endure larger sizes of the tumours compared to the smaller animals, then larger tumours may not grow indefinitely as hypertumour may appear during the cancer progression and reside in the tumour cluster [6].

As CGFs are the intermediary of cancer cooperation, numerous anticancer drugs such as bevacizumab (Avastin) [9], lapatinib (Tykerb) [10], erlotinib (Tarceva) [11] and sunitinib (Sutent) [12] target the growth factor or growth-factor-associated pathways and receptors. As a long-term adaptation to such therapy, however, cancer cells that produce larger amounts of CGFs or are resistant to the anticancer mechanisms might be selected for [13]. If the cancer is not totally eliminated, the remaining cancer cells are likely to be more resistant to the therapy. Once these cells proliferate, the relapsing cancer would be robust against the previous therapies. In short, therapy-resistant cancer cells become dominant after the bottleneck (clonal selection) [14], which is often overlooked in medical research [15].

**Figure 1.**
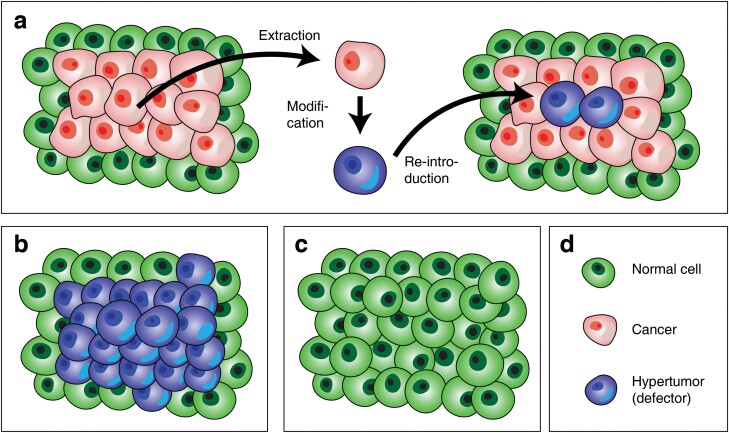
The illustration of the hypothetical ACD for cancer elimination. (a) Suppose that a cluster of cancer cells (red cells) resides in normal tissue (green cells). A cancer cell can be extracted and modified into a hypertumour cell that does not emit growth factors while taking benefits from other cancer cells. Those hypertumour cells grown *in vitro* can be implemented into the cancer cluster. (b) If the relative fitness of the modified hypertumours is higher than the existing cancer cells, the cancer cluster will be encroached on by the hypertumour cells. (c) If the hypertumours are not self-sustaining, then the hypertumour cluster will collapse, replaced by adjacent normal cells. (d) The graphical legends of the cell types.

As a group of cooperative agents is susceptible to selfish defectors [16], the artificial introduction of defectors into the tumour cluster may demolish the stability of the group [17]. In other words, one may plant artificially generated hypertumour to the tumour cluster. Rather than enforced elimination of cancers, inducing the collapse of the group integrity by introducing defectors may be an efficient method of cancer elimination [17]. To embody this method, cells from the cancer cluster should be extracted and modified so that they do not produce CGFs. Then, they could be implemented in the cancer cluster, working as a hypertumour (autologous cell defection (ACD) seusu Archetti [17], Fig. 1). To provide intuition and motivation to medical practitioners, I developed a simulation model that describes the progression of the cancer cells within normal tissue in the presence or absence of hypertumours. In this simulation, cancer cells emit three kinds of diffusible CGFs and acidifying lactate (the by-product of the Warburg effect) that damages the normal cells [3, 18]. The influence of the anticancer drugs that reduce the effect of specific CGFs also came into account.

To further investigate the effect of the anticancer drugs on ACD, the fitness of the cells adjacent to the hypertumour–cancer interface was examined in a deterministic manner under different assumptions on the fitness functions. The stochastic spatial simulations and deterministic analyses clarify the directions and caveats for the efficient clinical implementation of ACD.

## SIMULATION MODEL FORMULATIONS

To illustrate cancer emitting diffusible growth factors, three CGFs were assumed. Acidifying lactate was also considered embracing the influence of the Warburg effect. Diffusible factors promote the growth of the adjacent tumour cells (including the producer itself) with the corresponding mutated receptors [19]. Lactate damages the cells that do not have resistance to acidification [3, 18]. Secretion of the CGFs imposes the cost (reduction of fitness). Each cell has fitness, the capability to replicate when the nearby cell (in the von Neumann neighbourhood) is deceased (death–birth process [20]). Cells with higher fitness are more likely to prosper.

In each time step, mutation may occur in the alleles of each cell. There are four genes that are responsible for the secretion of three CGFs and lactate. Mutations in those genes make the cell produce such diffusible factors. Genes responsible for the specific growth factor receptors should be mutated to gain considerable fitness advantage from the growth factors. Mutation in another gene confers resistance to lactate. Highly mutated cells are more likely to be detected by the immune cells and eliminated [21] while there also is a gene at which the mutation allows the cell to evade such immunity in this simulation as the immune system often responds erroneously [22]. The description of the oncogenes in the simulation is presented in Supplementary Table S1.

Here, it was postulated that a cluster of cancer cells endowed with full hallmarks of cancer [5] is present. This would be the consequence of the metastasis where a fully transformed cancer migrates and establishes a cluster. As a novel treatment for cancer, the tumour cells can be extracted and then modified to be defectors of the cancer. The modified defectors (hyptertumour) should take advantage of CGFs (with the corresponding receptors), and have resistance to the lactate, but should not produce CGF.

**Figure 2.**
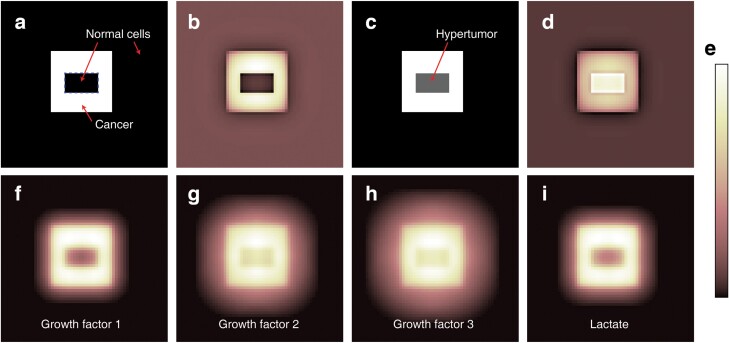
The initial condition of the simulations of Model A. (a) The spatial distribution of the normal (black grids) and cancer cells (white grids) to estimate the dynamics of the cancer progression. A cancer cluster is located at the middle of the normal cells. The centre of the cancer cluster (marked with a dashed blue line) is filled with normal cells. (b) The fitness map of (a). The brighter colour indicates higher fitness (legend is shown in (e)). (c) The spatial distribution of the normal, cancer cells and hypertumours (grey grids). The hypertumour that does not exhibit the Warburg effect is located at the centre of the cancer cluster. (d) The fitness map of (c). Hypertumour cells have higher fitness than adjacent cancer cells. (f–i) The concentration of the hypothetical growth factors 1 to 3 ((f) to (h)) and the lactate (i) generated by the cancer cells in (a) or (c). Growth factor 1 has the shortest diffusion range among the growth factors, while growth factor 3 has the longest range. The concentration of the lactate may differ if the hypertumours exhibit the Warburg effect.

To expect the effect of the defector injection, a testbed composed of 100 × 100 cells was established. In the middle of the testbed, a rectangular-ring-shaped 22 × 22 cancer cluster was located. At the centre of the cluster, 12 × 7 modified defective hypertumours exhibiting the Warburg effect were implemented (Fig. 2). Additionally, another type of hypertumours that does not exhibit the Warburg effect was tested. As control simulations, the centre of the cancer cluster was filled with normal (unmutated) cells. For three spatial configurations, the drug that reduces the effects of the CGF 1 and 2 by half was applied or not to estimate the influence of the anticancer drugs during ACD. To reduce complexity, it was assumed that the CGF has a positively linear effect on the fitness of the cells with the corresponding receptors.

**Figure 3.**
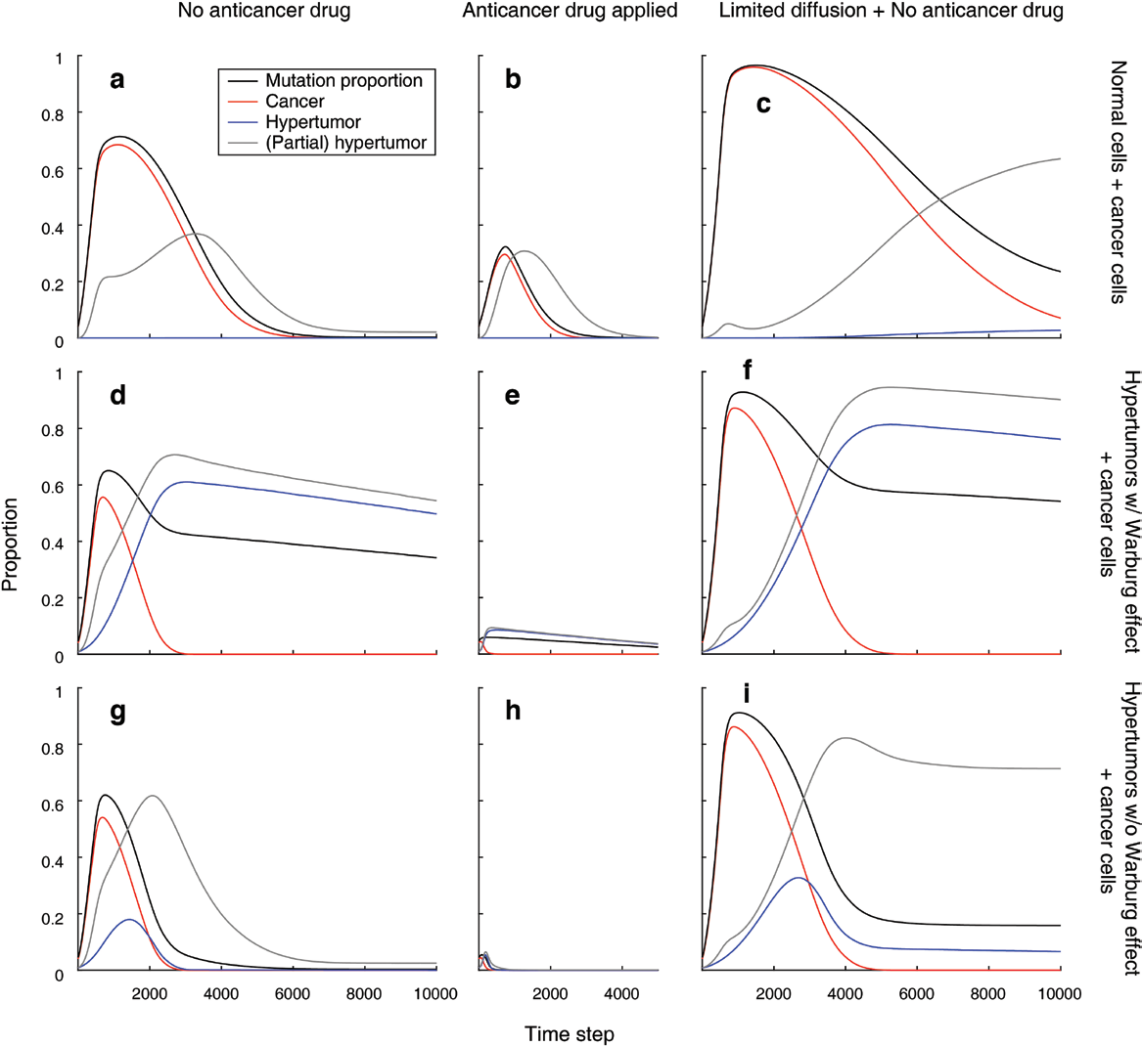
The dynamics of the cell proportions in diverse initial conditions. (a) The dynamics of the cancer propagation in Model A (intermediate diffusion range) when there is no hypertumour at the beginning of the simulation as in Fig. 2a. The cancer cells propagate at the early stage of the simulation while they later regress due to the spontaneously emerging partial hypertumors. ‘Mutation proportion’ represents the proportion of the mutated alleles of the cancer-related genes. ‘(Partial) hypertumour’ indicates the sum of the hypertumour and the partial hypertumour. (b) Same as (a) when an anticancer drug that reduces the effect of the CGFs 1 and 2 by half is applied. Unlike the assumption of the non-linear relation used in the numerical calculation (Fig. 5), the effect of the cancer growth factor on fitness was assumed to be linear. In this case, the anticancer drug reducing the CGFs effectively impedes the cancer progression. (c) Same as (a) when the cancer cells secrete growth factors with a short diffusion range but the average concentration within the diffusion range is higher (Model B). (d–f) Same as (a–c) when the centre of the cancer cluster is filled with hypertumour as in Fig. 2c. In this condition, the hypertumour exhibits the Warburg effect. Though cancers become extinct, remaining hypertumours persist. (g–i) Same as (a–c) when the centre of the cancer cluster is occupied by the hypertumours that do not exhibit the Warburg effect. Unless the growth factor diffusion range is short and concentrated, hypertumours of this type without cancer cells are replaced by normal cells, the ideal dynamics of the ACD.

Five hundred replications were performed for each condition (three spatial configurations and application of the drugs). The simulation lasted for 10 000 time steps, and in each time step, the proportions of the cancer cells (with full hallmarks of cancer) and the hypertumours were counted. The average mutation proportion calculated as the proportion of the mutated alleles of the oncogenes was also measured. While hypertumours are responsive to all CGFs, some cells that do not produce any CGF may not be responsive to all CGFs. Here, partial hypertumour was defined as the cells that do not emit CGF but take advantage of 1 or 2 (but not 3) CGF(s). The proportion of partial hypertumours was also counted (Fig. 3, Supplementary Figs. S2–S7).

To test the effect of the permeability of the tissue microenvironment, three models with different diffusion ranges of the tissue were simulated (Models A, B and C). Higher permeability increases the diffusion range but instead lowers the average concentration within the diffusion range so that the total sum of the concentration values within the diffusion ranges is conserved for the same CGF regardless of the Models. A CGF has the shortest range in Model B and the longest range in Model C. The visual description of the CGF diffusion is shown in Supplementary Information (Supplementary Fig. S1). The simulation lasted for 30 000 time steps for Model B when no anticancer drug was applied in order to observe the finalized configuration. As the final composition of Model B when no anticancer drug was applied is highly divergent, the dynamics when the final proportion of the (partial) hypertumour (which indicates the sum of the hypertumours and the partial hypertumours) is above 0.5 or below 0.5 were separately analysed (Supplementary Figs. S6 and S7).

## MATHEMATICAL MODEL FORMULATIONS FOR THE EFFECT OF ANTICANCER DRUG

**Figure 4.**
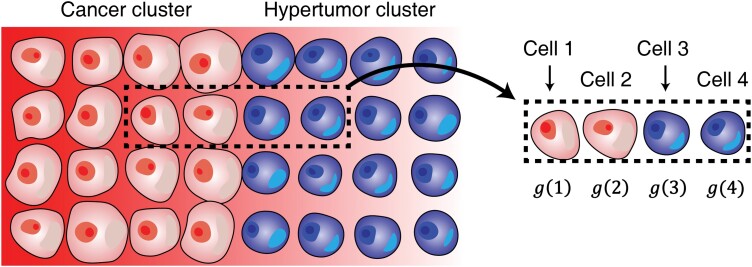
The basic schematics of the numerical calculation to estimate the effect of the growth-factor-reducing anticancer drug on hypertumour propagation. It was assumed in the mathematical model (not the simulation model) that there is a large cluster of cancers that is adjacent to the large cluster of the hypertumours. Due to symmetry, the growth factor concentration would be solely dependent on the displacement from cancer–hypertumour interface shown as the red horizontal gradient. To analyse whether hypertumours will propagate, the fitness of four cells near the border (marked with a dashed line) can be compared. The growth factor concentration values of those four cells were named *g*(1) to *g*(4) that determine the fitness of the respective cells.


In addition to the simulation model, I built a simple mathematical model that is focussed on the progression of the hypertumour cells at the hypertumour–cancer interface. On the assumption that a deceased cell is replaced by a progeny of an adjacent cell, four cells near the interface were taken into account. The effect of the CGF on the fitness of cancer or the hypertumour cells could be non-linear, often assumed to be logistic [23]. Suppose that a square-shaped layer of cancer cells is adjacent to the square-shaped layer of the hypertumour cells (Fig. 4). Define Cell 2 as a cancer cell that is adjacent to the hypertumour cell (Cell 3). Also, define Cell 1 as the cell that is adjacent to Cell 2 and interior to the cancer cluster. Cell 4 is a hypertumour cell adjacent to Cell 3 and interior to the hypertumour cluster as shown in Fig. 4. Define g(1) to g(4) as the concentration of the CGF at Cell 1 to 4. As the CGF is released from the cluster of cancers, the following will hold.

$$\begin{array}{l} \begin{array}{l} g(1)>g(2)>g(3)>g(4).\; \end{array} \end{array}$$ (1)

For simplicity, assume that the function of the fitness gain from the specific CGF concentration is the same for the tumour and hypertumour. The fitness of the four cells in the model is


$$\begin{array}{l} \begin{array}{l} \omega_{1}=F(g(1))-c,\; \end{array} \end{array}$$ (2)

$$\begin{array}{l} \begin{array}{l} \omega_{2}=F(g(2))-c,\; \end{array} \end{array}$$ (3)

$$\begin{array}{l} \begin{array}{l} \omega_{3}=F(g(3)),\; \end{array} \end{array}$$ (4)

$$\begin{array}{l} \begin{array}{l} \omega_{4}=F(g(4))\; \end{array} \end{array}$$ (5)

where F is the CGF-to-fitness transformation function, $\omega_{i}$ is the fitness of the i th cell, and *c* is the cost of producing the CGF. *F* should be a monotonically increasing function.

Suppose that Cell 1 is removed. Assume that the removed cell is replaced by one of the adjacent cells at the same row (linear spatial structure). Then Cell 1 will be replaced by the cancer cells, which does not affect the total proportion of the cancer cells. Suppose that Cell 2 is removed. Depending on the fitness of Cell 1 and Cell 3, that cell will be replaced by a cancer cell or a hypertumour cell as both types of cells are adjacent to Cell 2. The same principle applies to the replacement of Cell 3. Hence, for hypertumours to stably proliferate in the cancer cluster, the following conditions should hold.


$$\begin{array}{l} \begin{array}{l} \omega_{1}<\omega_{3}\;and\;\omega_{2}<\omega_{4}.\; \end{array} \end{array}$$ (6)

Take ${\omega^{\prime }}_{1}$ to ${\omega^{\prime }}_{4}$ as the fitness of the Cell 1 to 4 after the application of the anticancer drug which reduces the CGF concentration to rg(i) (0<r<1). Then, $\;\Delta \omega_{i}$ can be defined as the change in fitness caused by the application of anticancer drugs ($\;\Delta \omega_{i}={\omega^{\prime }}_{i}-\omega_{i}$).

For anticancer drugs that target the CGF or the associated pathways to promote the progression of the hypertumours, the following conditions should hold.


$$\begin{array}{l} \begin{array}{l} \Delta \omega_{1}<\Delta \omega_{3}\;and\;\Delta \omega_{2}<\Delta \omega_{4}.\; \end{array} \end{array}$$ (7)

These conditions are equivalent to the following conditions.


$$\begin{array}{l} \begin{array}{l} F(rg(1))-F(g(1))<F(rg(3))-F(g(3)),\; \end{array} \end{array}$$ (8)

$$\begin{array}{l} \begin{array}{l} F(rg(2))-F(g(2))<F(rg(4))-F(g(4)).\; \end{array} \end{array}$$ (9)

Suppose that function F is linear so that F(t)=At+B for some constants A (A>0) and B as in the simulation in this study.


$$\begin{array}{l} F(rg(1))-F(g(1))<F(rg(3))-F(g(3)) \end{array}$$

$$\begin{array}{l} ↔(r-1)Ag(1)<(r-1)Ag(3) \end{array}$$

$$\begin{array}{l} \begin{array}{l} ↔g(1)>g(3).\; \end{array} \end{array}$$ (10)

By the same principle, $\;\Delta \omega_{2}<\Delta \omega_{4}\;↔g(2)>g(4)$. Hence, anticancer drugs that proportionately reduce the CGF promote the progression of the hypertumours if the effect of the CGF is linear and conditions of Inequalities [6] are satisfied.

**Figure 5.**
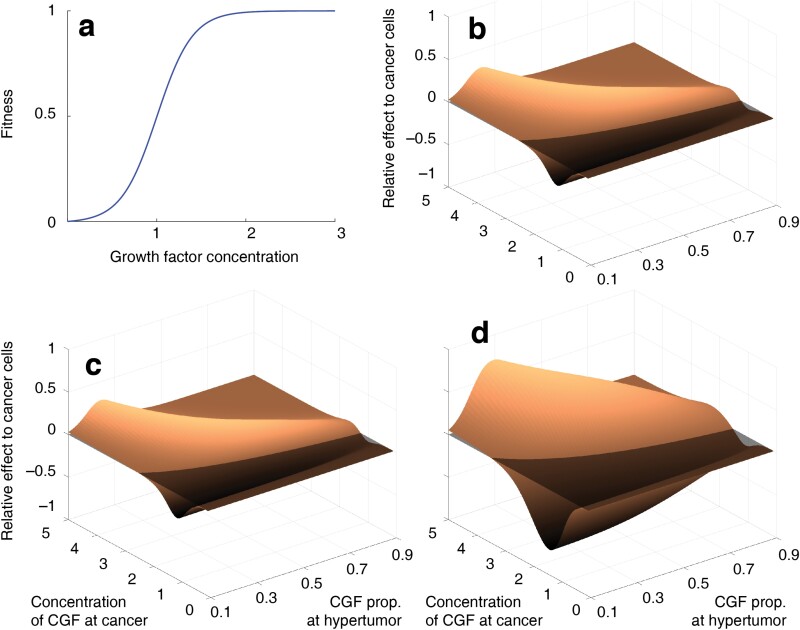
The results of the numerical computations based on the mathematical model analysing the fitness at the cancer–hypertumor interface. (a) In this computation, the growth-factor-to-fitness transformation function was assumed to be logistic. (b) The relative effect on the cancer cells which is calculated as the fitness change of the cancer cells induced by the anticancer drugs (reducing the growth factor effect) subtracted by that of the hypertumour cells. The lower these values are, the more likely that the hypertumour progression is promoted. The *y*-axis represents the concentration of the growth factors in the cancer cells (i.e. *g*(1) or *g*(2)). *x*-axis represents the proportion of the growth factors in the hypertumors compared to the cancer cells (i.e. *g*(3)/*g*(1) or *g*(4)/*g*(2)). This value should be lower than 1 as the growth factors in the hypertumour are lower than those in cancers. This is the result when anticancer drugs reduces the 20% of the CGFs (or equivalently disrupts the associated pathways). (c) Same as (b) when anticancer drugs reduces 50% of the CGFs. (d) Same as (b) when anticancer drugs reduces 80% of the CGFs.


For non-linear F, it is difficult to analytically estimate the general effect of the anticancer drugs. For example, if F is a logistic function, anticancer drugs can exhibit opposite effects depending on the shape of F and the CGF concentration. I postulated a hypothetical logistic function (Fig. 5a) in the following structure.

Undefined control sequence \vspace (11)

where $k_{0}=1.0067$, $k_{1}=5$ and $k_{2}=-0.0067$ so that F(0)=0 and $\underset{t\to \infty }{\lim}\;F(t)=1$.

The relative effect of the anticancer drugs on the cancer cells (*z*-axis of Fig. 5b–d) was calculated as $\phi =\Delta \omega_{1}-\Delta \omega_{3}$ or $\;\Delta \omega_{2}-\Delta \omega_{4}$. If ϕ is negative, then application of the anticancer drugs promotes the hypertumour progression at the hypertumour–cancer interface (Refer to Inequalities [7]).

I changed the CGF concentration in the cancer cells and the hypertumour cells with the different intensities of the anticancer drug effect to measure the fitness change of the two types of cells. In addition, identical sets of numerical calculations were performed for the convex and concave fitness functions (Supplementary Figs. S8–S10).

## RESULTS

In each simulation model, three initial conditions were tested under which the centre of the cancer cluster is filled with normal cells, hypertumours with the Warburg effect, or hypertumours without the Warburg effect. Dynamics of the cell progression were measured with or without the application of the anticancer drugs that reduce the effect of CGFs 1 and 2. Hence, for each simulation model, six result graphs can be obtained. To estimate the effect of the CGF diffusion, three different models with different CGF diffusion ranges were tested (Models A, B and C). Model A has the intermediate diffusion range and Model B has the shortest diffusion range (Supplementary Fig. S1). All simulation results are presented in Supplementary Figs. S2–S7.

In all cases, the (partial) hypertumours spontaneously appear as the result of the mutations. They can emerge if oncogenes of the normal cells responsible for the CGF receptors (or the related pathways) are mutated while such mutation does not occur in the oncogenes for the CGF production. The existence of the (partial) hypertumours, as a result of the implementation or spontaneous emergence, and the corresponding increment of them leads to the regression of the cancer cells given that the normal cells are not extinct. In all simulation models, the introduction of the hypertumours at the initiation of the simulation accelerated the regression of the cancer cells. Once cancer cells on which (partial) hypertumours are reliant became minuscule or extinct, (partial) hypertumours subsequently regressed. However, if the proportion of the (partial) hypertumour reaches 1, then such proportion is fixated (Supplementary Fig. S6) as the reverse mutation (the mutation that restores the normal function of the cell) was not postulated in this simulation: normal cells can be produced only by replication of the normal cells.

The (partial) hypertumours can gain a higher fitness advantage when the proportion of cancer cells on which they are parasitic is high. Short yet concentrated CGF diffusion promotes the cancer progression, during which (partial) hypertumour can abruptly prosper (Supplementary Fig. S3). In contrast, the wider range of the CGFs accompanied by the low average concentration within the range impeded the cancer progression (Supplementary Fig. S4), which is supported by the comparison of Models A, B and C. Hypertumours exhibiting the Warburg effect regressed slowly compared to those that do not exhibit the Warburg effect. On the assumption that CGFs have a linear effect on cell proliferation, the application of the anticancer drug that reduces the CGF effect exhibited therapeutic results. The examples of the simulations are presented in Animations S1–S18.

In addition to the simulation results, I developed a simple model that illustrates the fitness of four cells at the hypertumour–cancer interface. CGF concentration levels at the hypertumours and the cancer cells were varied to estimate the progression of the hypertumour. The influence of the anticancer drugs with different efficacy was tested. The effect of the anticancer drugs was variable depending on the schematics of the fitness functions. The numerical calculation results show that anticancer drugs may disturb ACD if there is a strong concavity in the fitness function (Supplementary Figs. S8–S10).

## DISCUSSION

The conventional paradigm of cancer treatment is generally focussed on the elimination of cancer, which becomes the basis of injecting anticancer drugs with ‘maximum tolerable dose [24]’. This strategy is seemingly straightforward while it was pointed out that the evolutionary perspective of treatment should be considered to prevent the relapse of the cancer [15]. An alternative strategy to this ‘unconditional annihilation’, metronomic chemotherapy characterized as the low administration of anticancer drugs for an extended period was proposed as a safer way of treatment with alleviated side effects though its advantage is not conclusive [25]. As a ‘cunning’ strategy to interfere with the integrity among the altruistic cancer cells, ACD was suggested to treat cancer by which tumour cell is modified into the defectors of the ancestral tumour cells [2, 3, 17, 26]. These parasitic hypertumour cells will lead to the ‘tragedy of the commons’, disrupting the cooperation among the tumour cells [23, 27]. For tumour cells, the common goods are the growth factors or lactate that are beneficial to respective individuals of the specific types (cancers) while demanding the cost of production [23, 27]. Recently, the applications of the game theory to empirical experiments with tumour cells were performed [26, 28], shedding light on the medical application of ACD for cancer treatment.

**Figure 6.**
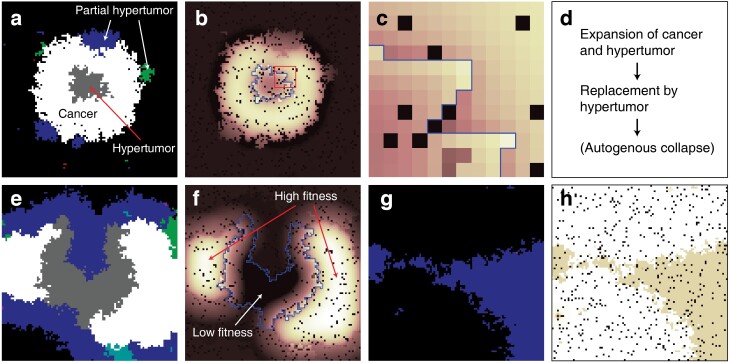
The example dynamics of the ACD. (a) Hypertumours without the Warburg effect were implemented in the centre of the cancer cluster as in Fig. 2c. This is the snapshot of the cell arrangements at time step 260. Grey cells in the centre are the hypertumours without the Warburg effect. Green and blue cells are the spontaneously generated partial hypertumours responsive to growth factor 2 and growth factor 3, respectively. White cells are cancers, and black cells are normal cells. (b) The fitness map of (a). The boundary of the hypertumour cluster was marked with a blue line. The sparsely distributed black grids are the deceased cells. (c) The magnified fitness map of (b) marked with a red square. The fringe of the hypertumour cluster exhibits higher fitness due to the growth factors emitted from the adjacent cancer cells. In contrast, the fringe of the cancer cluster exhibits lower fitness due to the low growth factor concentration compared to the concentration in the centre of the cancer cluster. (d) The process of hypothetical ACD for cancer elimination. (e and f) Same as (a) and (b) for time step 1 100. Hypertumours are replacing cancer clusters. Teal grids represent partial hypertumour cells responsive to growth factors 2 and 3. The centre of the hypertumour cluster (marked with the blue line in (f)) exhibits low fitness, while the centres of the cancer clusters (white clusters in (e)) exhibit high fitness. (g and h) Same as (a) and (b) for time step 3 000. All cancer cells were replaced by partial hypertumours. It was assumed in the simulation model that mutated cells exhibit lower fitness unless they receive benefits from the growth factors. Hence, hypertumours without cancer cells are replaced by normal cells if they do not exhibit the Warburg effect.

The process of ideal hypothetical ACD treatment should be composed of (i) progression of both tumour and hypertumour cells; (ii) encroachment of the cancer cluster by hypertumours; (iii) collapse of the hypertumour (Fig. 6d). The simulation results show the feasibility of ACD and the required conditions for successful treatment. Most of all, it is ideal if the progression speed of the defective hypertumour is greater than that of the existing cancer. Although the growth of the defective hypertumour is slower than the cancer cells, if the implementation of the hypertumour impedes the growth of the cancer cluster, ACD may have clinical significance.

The mathematical computation results in this study (Fig. 5, Supplementary Figs. S8–S10) and the previous empirical results [26] indicate that the relative fitness of cancer and hypertumour is dependent on the ambient growth factor concentration. Due to the complicated effect of the anticancer drugs on hypertumour progression (Fig. 5), the application of the anticancer drug targeting the CGF during ACD should be discreetly decided. Suppose that the growth-factor-to-fitness function is logistic and the CGF of the cancer cells is located at the plateau of the logistic fitness function so that a change of such CGF concentration does not alter the fitness significantly. On the other hand, suppose that the CGF of the hypertumour is located near the inflection point of the logistic fitness function where the change of the CGF considerably affects the fitness. In this case, the application of the anticancer drugs would have minimal effect on the cancer cells while dramatically reducing the fitness of the hypertumour cells, reducing the effect of the ACD. Generally speaking, CGF-reducing anticancer drugs may obstruct ACD when the slope of the fitness function at the CGF concentration of the hypertumour is greater than the slope at the CGF concentration of the cancer. Given that the concentration of CGF at hypertumour is lower than that of cancer, the fitness function should exhibit concavity at a certain interval for anticancer drugs to obstruct ACD. The concavity of the fitness function does not always lead to the interruption of ACD when CGF-reducing anticancer drugs are applied. The counterexample of this case is shown in Supplementary Fig. S9.

The growth-factor-to-fitness function might be empirically measured with the terms of the microenvironment. For example, cancer-associated fibroblasts affect the growth rate of the cancer cells in the presence of anticancer drugs [28, 29]. The experimental results indicate that the effect of the IGF-II concentration on the cancer growth rate might be concave [27]. If the marginal effect of the growth factor on fitness is decreasing (yet positive) as the growth factor concentration increases, the fitness function would be concave. Given that there is a limitation of the growth rate, additional provision of growth factor would have a negligible effect when the growth rate is near the upper bound. Then the growth rate against the growth factor around that domain could be concave. Such concavity in growth rate function is found in microorganism growth against the nutrient concentration [30, 31]. Once the relationship between the growth factor and the relative fitness is elucidated, then one would be able to decide whether the application of the anticancer drug is beneficial or detrimental to ACD.

Although numerous models analysing ACD were presented [2, 3, 17, 26], most of the models assumed well-mixed situations presumably due to handiness. However, spatial distribution is crucial for cancer dynamics [29, 32, 33], especially considering that like-with-like assortment with co-operators promotes the co-operator fitness [34]. As cancer cells emit diffusible factors influencing the adjacent cells, spatial arrangements should be the pivotal factor in cancer progression studies. Though simplified spatial simulation models about ACD were previously conducted [17, 23], there has been no model that embraced the multiple growth factors and the acidifying lactate with different diffusion ranges (Fig. 2f–i). As tumours in the actual human or animal body exhibit rigid structures and multiple CGFs are involved, the simulation model considering the spatial framework would be more biologically realistic. Due to the spatial configuration of the cells and diffusible CGFs in this simulation, the hypertumour cells that are located at the periphery of the hypertumour cluster facing the CGF-emitting cancer cluster exhibited higher fitness among the cluster (Fig. 6b,c,f). On the contrary, cancer cells at the periphery of the cancer cluster exhibited lower fitness among the cluster (Fig. 6b,c,f) due to less exposure to CGFs. The prominent trait of the simulation results considering the spatial structure is that the hypertumours may propagate although their average fitness is lower than the cancer cells. This is probable if the hypertumours locally dominate the cancer cells at the hypertumour–cancer interface as the deceased cell is replaced by one of the spatially adjacent cells (Fig. 6c). In a well-mixed model, it would be difficult for hypertumours to propagate if their average fitness is lower than other types of cells.

Short-range yet highly concentrated CGF will promote the progression of the hypertumours that are close to the hypertumour–cancer interface although the hypertumours that are remote from the interface cannot receive such an advantage. It is the fitness of the cells close to the hypertumour–cancer interface that determines the progression of the hypertumours given that each cell is replaced by the adjacent cells. Additionally, short-range CGF is an effective measure of nepotistic cooperation among the cancer cells leading to accelerated cancer progression. Meanwhile, an increased number of cancer cells supply more CGFs to the hypertumours. Dynamics shown in Supplementary Fig. S6 illustrate the saturation of the (partial) hypertumours followed by the upsurge of the cancer cells. In contrast, long-range yet sparsely concentrated CGF would elevate the fitness of the hypertumours that are remote to the hypertumour–cancer interface that has a minor effect on the hypertumour progression. Therefore, the most efficient method to enhance the hypertumour progression would be to blend the cancers and hypertumours so that most hypertumours are close to CGF-producing cancer cells.

Warburg effect itself is speculated to confer an advantage to the mutated cells over the normal tissue [18]. Hence, defective hypertumours exhibiting the Warburg effect may not collapse immediately after the extinction of the cancers as shown in simulation results. Consequently, ideal hypertumours for ACD should be modified not to exhibit the Warburg effect while they should be resistant to the lactate produced by cancer cells.

The microbial study of Kümmerli *et al.* [35] illustrates the effect of the environmental viscosity (correspondent to the tissue permeability in the simulation) on the fitness of the cells that produce altruistic diffusible factor. The higher viscosity of the medium (correlated with agar concentration) impedes the diffusion of the altruistic growth factor siderophore produced and utilized by *Pseudomonas aeruginosa* [35]. Increased medium viscosity resulting in limited diffusion elevated the fitness of the siderophore-producing (altruistic) cells compared to that of defecting cells that do not produce the siderophore [35]. This empirical finding is in the same line with the simulation results of the present study in that reduced diffusion of the CGFs promotes the spread of the cancer cells (producers of the altruistic CGFs). However, defectors that are close to the altruistic producers of the short-range diffusible factor will receive intensive benefits.

Production and diffusion of beneficial factors were pointed out as the method of microbial organisms to perform the narrow-sense kin discrimination (favouritism towards others with higher relatedness) that is based on spatial proximity [36]. As clones of a cell will be located in the vicinity of that cell, the short-range altruistic diffusible factor is more likely to elevate the fitness of the clones [36]. The long-range altruistic diffusible factor will also benefit distantly related cells including the hypertumours. The reduced concentration of such long-range factors due to the dispersion will exhibit a weakened effect on the self (reduced autocrine effect) and the nearby clones. A similar effect of the diffusion range in the spatial simulation for the cancer progression was previously reported [23]. Therefore, one may expect that medical therapy that expands the diffusion range with the corresponding reduction in the concentration will facilitate hypertumour progression. Additionally, anticancer drugs that target nepotistic short-range CGFs are expected to be more effective in preventing cancer progression given that there is no strong concavity in the fitness function.

Attention to ACD, especially in the empirical aspect, is unusually scarce. For cases where conventional cancer therapy is difficult to apply, ACD might be an alternative with minimal side effects. As the hypertumour cannot propagate indefinitely when detached from the CGF-producing cancer cells, segregated hypertumour cells that circulate around the body will not induce any ‘metastatic’ effect to the normal tissue unless a cluster of hypertumour with the Warburg effect is implemented to the normal tissue. In other words, hypertumour is parasitic only to the cancer cells, unlike the numerous anticancer drugs that damage normal tissue.

## Supplementary Material

eoad042_suppl_Supplementary_Tables_S1_Figures_S1-S10

## Data Availability

The simulation code and the supplementary animations are available at: https://zenodo.org/records/10065578
