# Supplementary material for: Spatial simulation of autologous cell defection for cancer treatment
Source: Evol Med Public Health. 2023 Nov 22;11(1):461–71. doi: 10.1093/emph/eoad042 (PMC10727474; doi:10.1093/emph/eoad042)
Supplement: eoad042_suppl_Supplementary_Tables_S1_Figures_S1-S10 [file eoad042_suppl_supplementary_tables_s1_figures_s1-s10.pdf]

Supplementary Information for:

Spatial simulation of autologous cell defection for cancer treatment

Jibeom Choi[1*]

[1] Department of Applied Mathematics, College of Applied Sciences, Kyung Hee University, Yongin 17104, Republic of Korea

*snu10@snu.ac.kr

Simulation description

It was assumed that 9 genes are responsible for the cancer progression of diploid organisms. Genes 1 and 2 are responsible for the production and responsiveness to the cancer growth factor 1 (CGF 1). Mutation of Gene 1 leads to the production of the CGF 1, while mutation of Gene 2 makes the cells become responsive to CGF 1. Responsiveness refers to the increased fitness (capability to replicate) when exposed to the specific CGF. Genes 3 and 4 are related to CGF 2, analogously to Genes 1 and 2. Similarly, Genes 5 and 6 are related to CGF 3. Production of the CGFs entails a cost to the cell, while the higher CGF concentration with the corresponding mutated receptor provides a fitness advantage to the cells. When Gene 7 is mutated, the cell is more likely to evade the immune response that eliminates the mutated cells. Mutation in Gene 8 confers the resistance to the lactate, the byproduct of the Warburg effect. Mutation in Gene 9 leads to the Warburg effect. The cells with this mutation produce lactate and have a cost in energy production. The role of each gene is presented in Table S1.

Table S1. Descriptions of the genes postulated in the simulation

Gene name	The function when the 2-hit mutation occurs
Gene 1	The cell produces cancer growth factor 1.
Gene 2	The cell expresses the receptor for cancer growth factor 1.
Gene 3	The cell produces cancer growth factor 2.
Gene 4	The cell expresses the receptor for cancer growth factor 2.
Gene 5	The cell produces cancer growth factor 3.
Gene 6	The cell expresses the receptor for growth factor 3.
Gene 7	The cell becomes more resistant to immune response.
Gene 8	The cell gains the resistance to the lactate.
Gene 9	The cell exhibits the Warburg effect and produces the lactate.

The simulation was performed on 100 × 100 grids (testbed). CGFs and lactate exhibit different diffusion ranges and different effects on the growth of other cells with the corresponding mutated receptors. It was assumed in the simulation that the order of the diffusion ranges is, from the highest to the lowest, CGF 3, CGF 2, lactate, and CGF 1. Three models with different tissue permeability were tested. Diffusion ranges of CGFs in Model B are the shortest among the three models, while the average concentration is the highest. Model C has

the opposite trend: Diffusion ranges of CGFs are the longest while the average concentration is the lowest (Figure S1). Across the models, the total amount of the CGFs or lactate that a cell contributes to the testbed is conserved as well as the order of the diffusion ranges. This indicates that if the diffusion range of a certain CGF becomes greater, the average concentration of the CGF within the diffusion range accordingly decreases.

In the middle of the testbed, fully mutated cancer cells (Genes 1 to 9 are fully mutated) are located in an angled-ring-shaped manner. At the center surrounded by fully mutated cancer cells, normal cells or hypertumor cells are located. Hypertumor cells have mutated receptors responsive to CGFs 1 to 3, are resistant to lactate, are evasive to the immune system. In other words, Genes 2, 4, 6, 7, 8 of hypertumor cells are mutated so that they do not produce CGF while taking advantage of CGFs produced by other cancer cells. Hypertumor cells may or may not exhibit Warburg effect (mutation of Gene 9). Partial hypertumors do not produce CGF but are responsive to 1 or 2 CGFs.

In every time step, each normal allele may mutate. If two alleles of a gene are normal, then the mutation rate was assumed to be 0.000003. Given that one allele is mutated, then mutation rate of another normal allele was assumed to be 3 times higher than mutation rate when both alleles are normal[1]. Reverse mutation, the mutation that restores the normal function of the cell, was not assumed. The application of the drug reduces the effect of the CGFs 1 and 2 into half.

Cells may randomly die or attacked by the immune cells. The higher proportion of the mutated alleles increases the probability to be eliminated by the immune cells. Mutation in Gene 7 (inspired by cancer’s evasion of immune response by not expressing major histocompatibility complex class 1[2]) reduces such probability of immune response. Deceased cells are replaced by adjacent cells, and cells with higher fitness are more likely to replicate.

The major variables of the MATLAB simulation code are explained in Table S2.

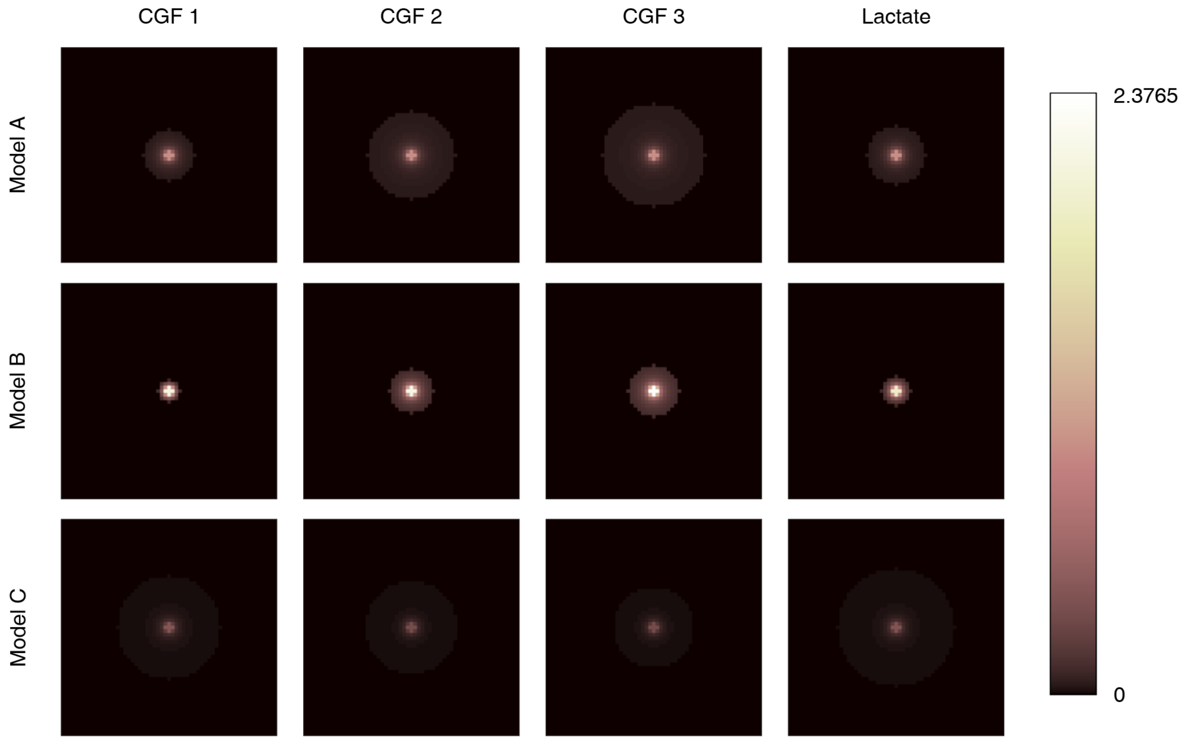

Figure S1. The schematics of the diffusion that a single cell establishes in different models.

A fully transformed cancer cell emits CGF 1 (cancer growth factor 1), GCF 2, GCF 3, and lactate with different diffusion ranges. In Model A, a moderate level of tissue permeability was assumed. Tissue in Model B has the shortest diffusion range, but the average concentration within the diffusion range is the greatest. Tissue in Model C has the highest permeability so that CGFs can spread to a wider range, but the average concentration within the range is the lowest. For each CGF, the total amount of the concentration on the grids is the same regardless of the models.

Table S2. The description of the major variables used in the MATLAB simulation code for the cancer progression.

Variable name	Description
DRG_App	If this value is 1, then anticancer drug is applied. If 0, then anticancer drug is not applied.
MID_HTM	If this value is 1, then the center of the cancer cluster is filled with hypertumors. If 0, then the center of the cancer cluster is filled with normal cells.
HTM_WAR	If this value is 1, then hypertumor cells in the center of the cancer cluster exhibits the Warburg effect. If 0, they do not exhibit the Warburg effect.
Tsteps	The number of time steps of the simulation.
Vis_On	If this value is 1, then progression is visually revealed. If 0, then graph is not revealed.
Show_intv	This value (a natural number) determines the interval to update the graph. For example, if this value is 50, then the graph is updated in every 50 time steps.
w_data	This 1×4 matrix contains the diffusion range of CGFs 1 to 3, and lactate.
drg_eft	This parameter (from 0 to 1) determines the effect of the anticancer drug. The lower this value is, the lower the effects of the GCFs 1 and 2 are.
ProdCost	This 1×3 matrix contains the production cost of CGFs 1 to 3.
AcidProdCost	This parameter contains the production cost of lactate (the byproduct of the Warburg effect).
AcidResistCost	This parameter represents the cost to gain resistance to the lactate.
AcidDam	This parameter determines the damaging effect of the lactate to other cells that lack the lactate resistance.
mu1	The probability that one allele of two normal homozygous alleles becomes mutated.
mu2	The probability that one allele becomes mutated given that another allele is mutated.
ImFidel	This value (from 0 to 1) explains the fidelity of the immune system. The higher this value is, the more likely that the immune system eliminates the mutated cells.

Detailed simulation results

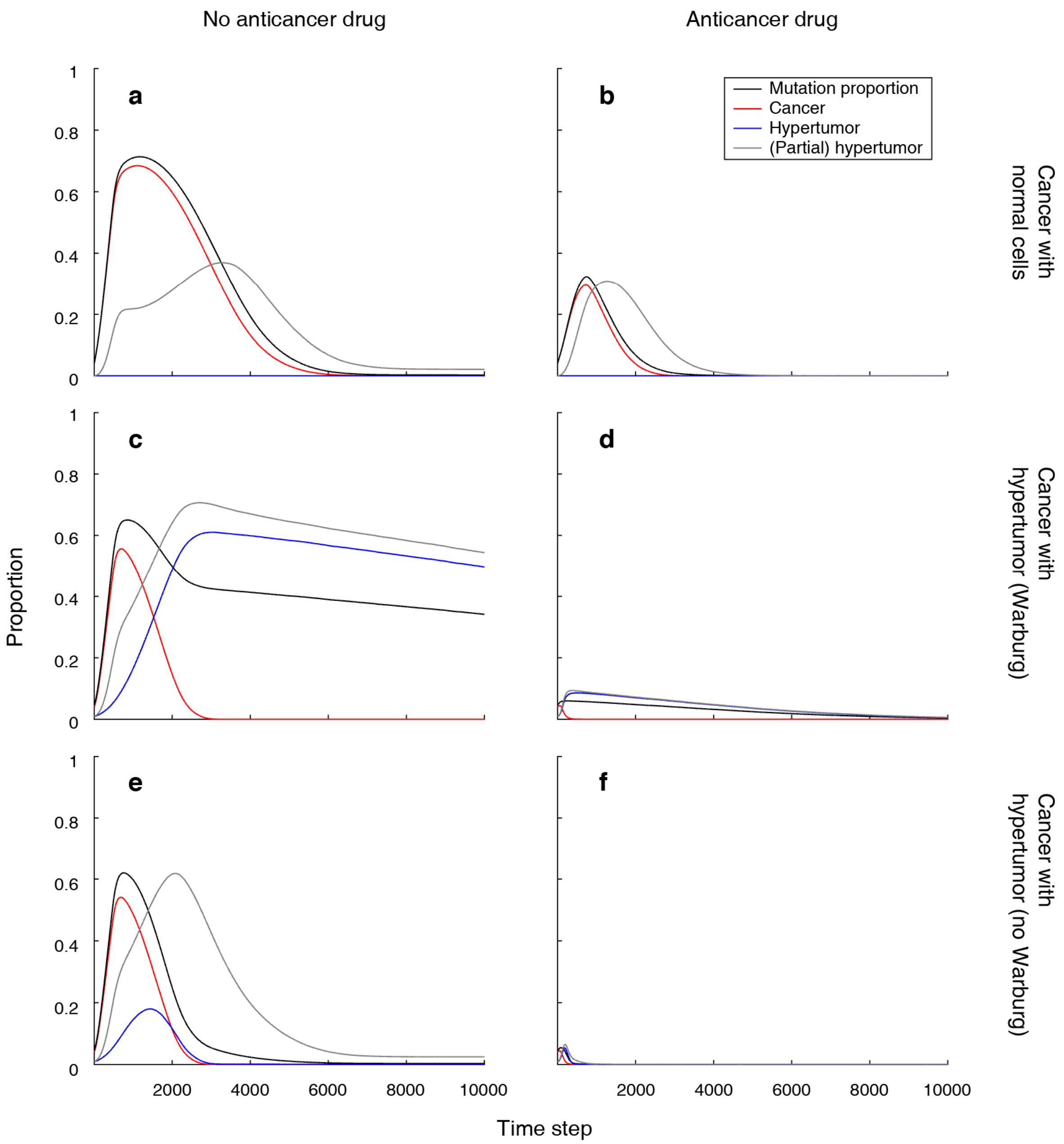

Figure S2. The proportions of different types of cells at each time step in Model A.

The diffusion range values of Model A are moderate compared to those of Models B and C. The graphs in the left column (panels (a), (c), (e)) represent the dynamics when the anticancer drug is not applied. The graphs in the right column (panels (b), (d), (f)) are the results when an anticancer drug is applied. The first row is the dynamics of the cancer progression in normal tissue (Figure 2a). The second row illustrates the dynamics when the center of the cancer cluster is filled with the hypertumors exhibiting the Warburg effect. The third row illustrates the dynamics when the center of the cancer cluster is filled with hypertumors that do not exhibit the Warburg effect. The inset of (b) explains the colors used in the graphs. '(Partial) hypertumor' represents the proportion of the hypertumors and the partial hypertumors.

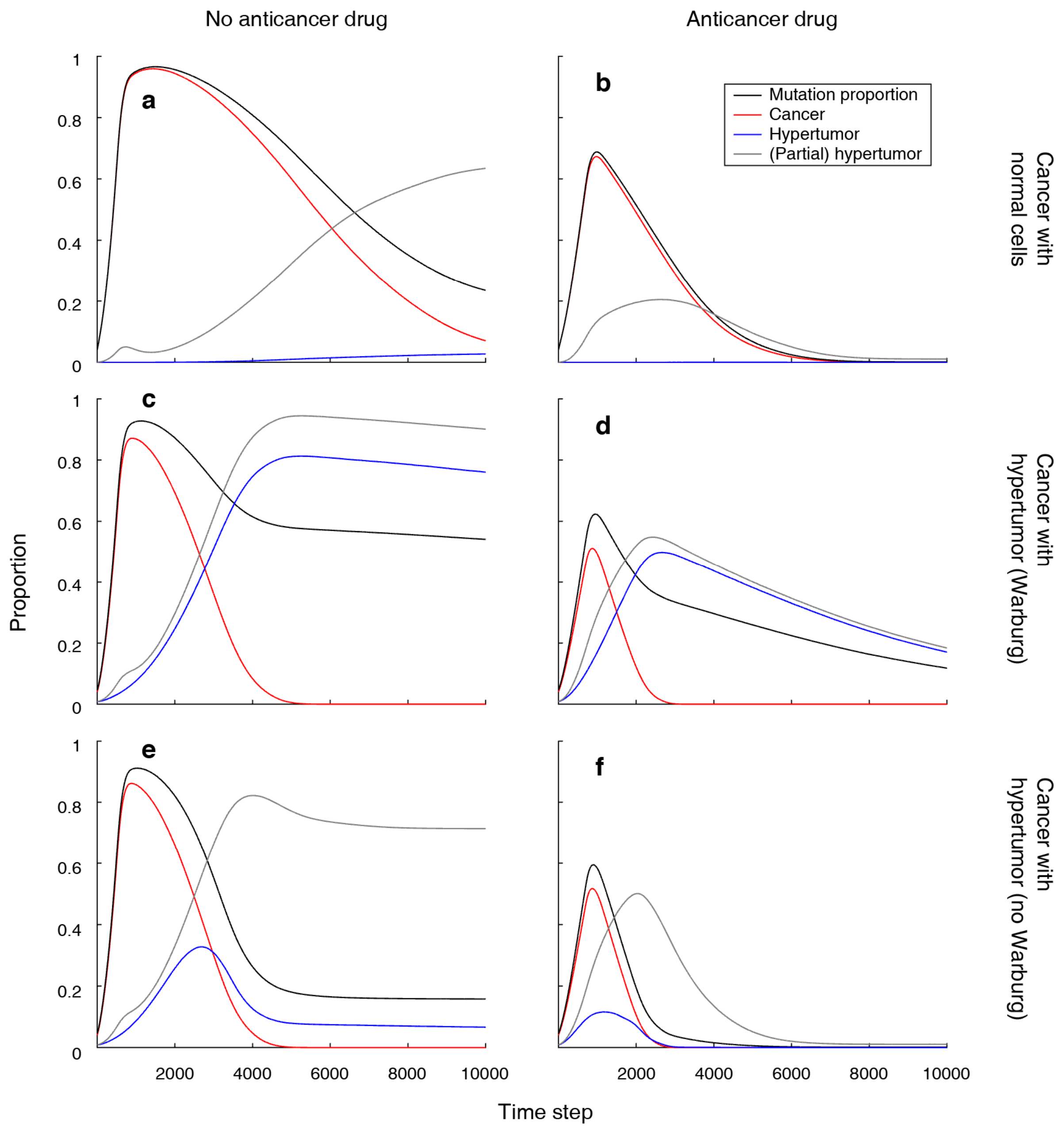

Figure S3. The proportions of different types of cells at each time step in Model B.

The diffusion range values of Model B are the shortest while the average concentration is the highest. The panels represent the same information as in Figure S2. As the dynamics of the left column are not clearly observable with 10,000 time steps, those simulations were performed for the 30,000 time steps whose results are revealed in Figure S5, Figure S6, Figure S7.

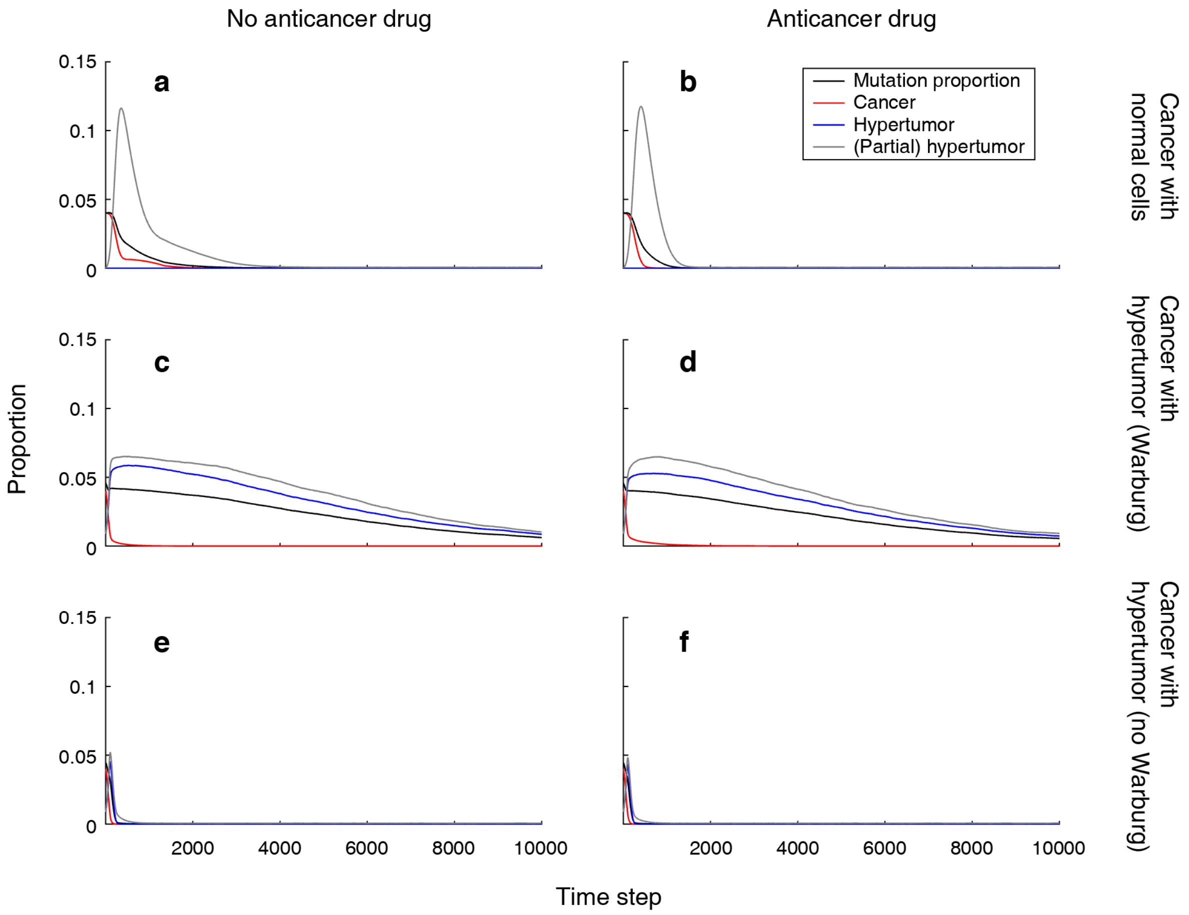

Figure S4. The proportions of different types of cells at each time step in Model B.

The diffusion range values of Model C are the longest while the average concentration is the lowest. Note that the scale of the *y*-axis is different from those of Figure S2 and Figure S3.

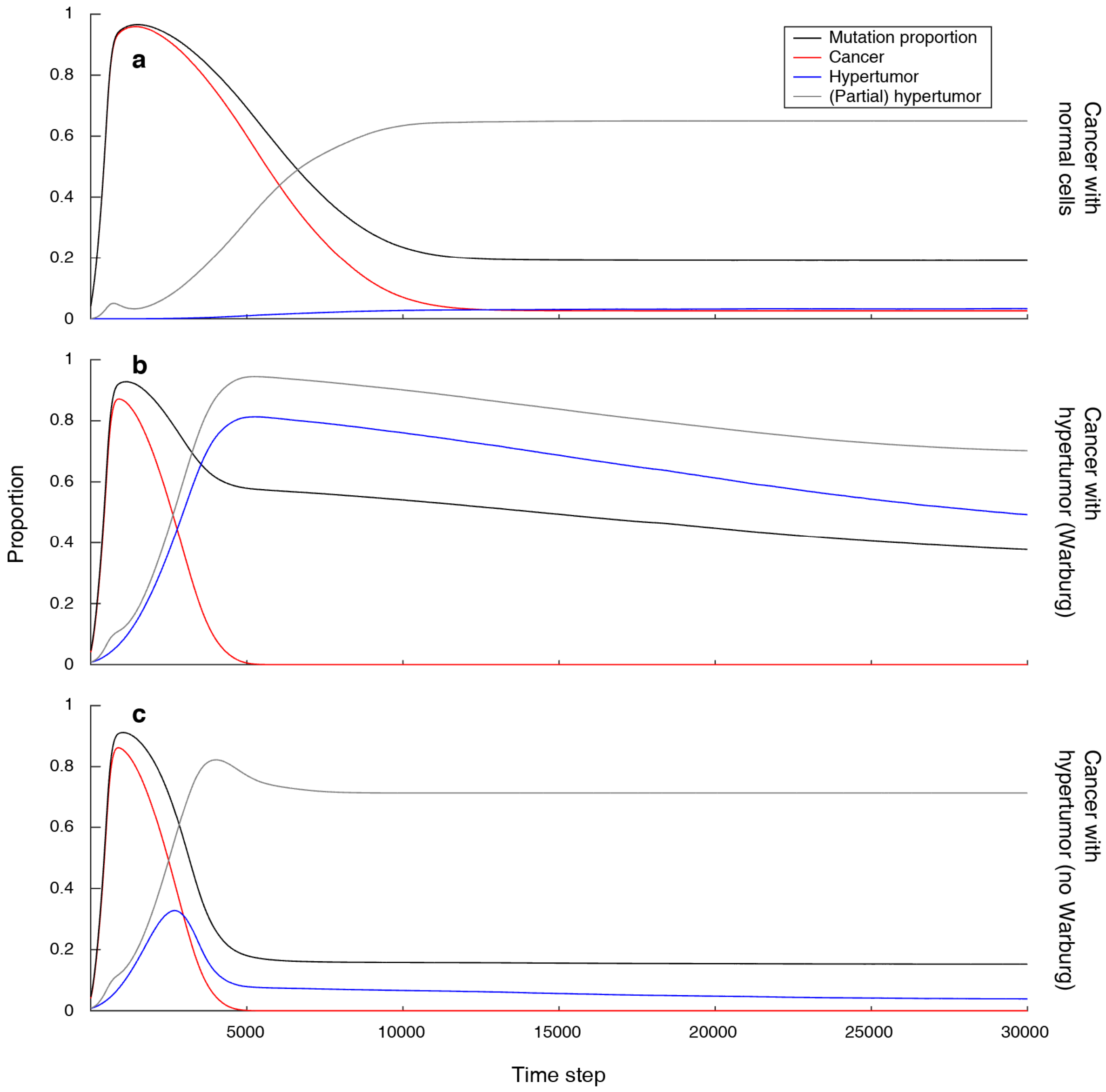

Figure S5. The simulation results of Model B for 30,000 time steps.

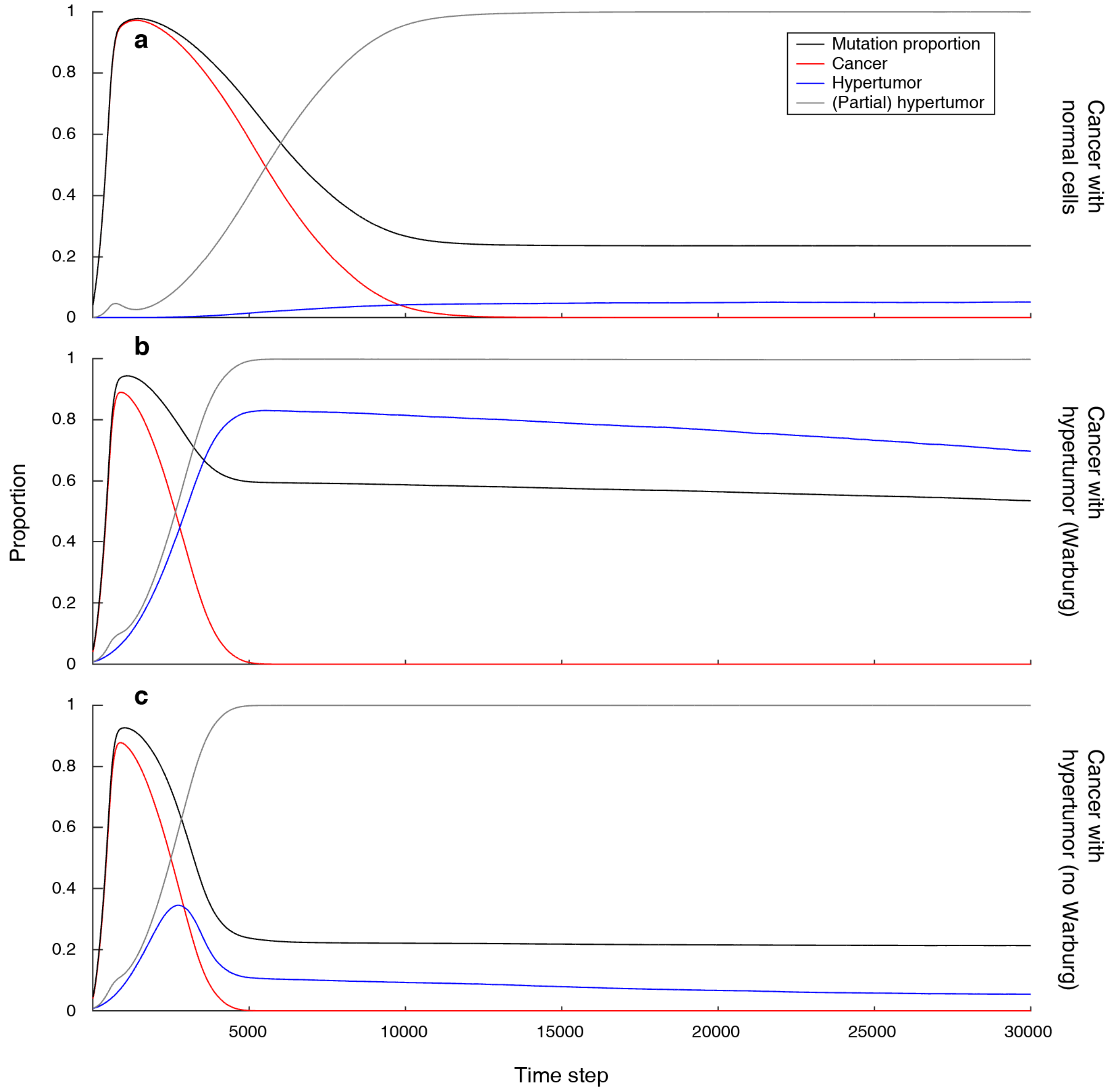

Figure S6. The simulation results of Model B in which the proportion of the (partial) hypertumor at the time step 30,000 is higher than 0.5. Out of 500 repetitions, 325, 348, and 357 cases for (a), (b), and (c), respectively, satisfied such (partial) hypertumor conditions at the end of the simulations.

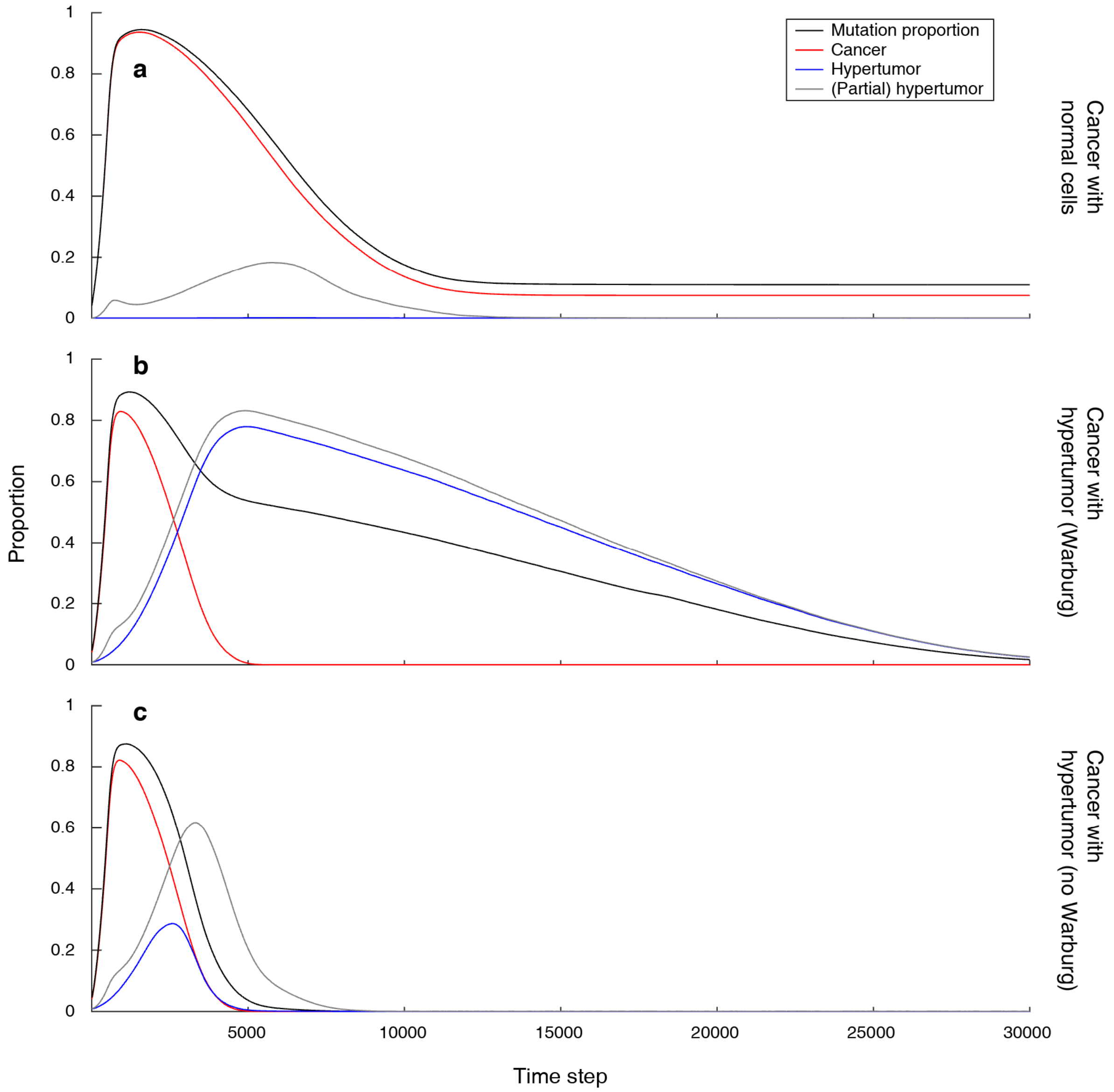

Figure S7. The simulation results of Model B in which the proportion of the (partial) hypertumor at the time step 30,000 is lower than 0.5. Out of 500 repetitions, 175, 152, and 143 cases for (a), (b), and (c), respectively, satisfied such (partial) hypertumor conditions at the end of the simulations.

Mathematical model for the anticancer effect at the hypertumor–cancer interface

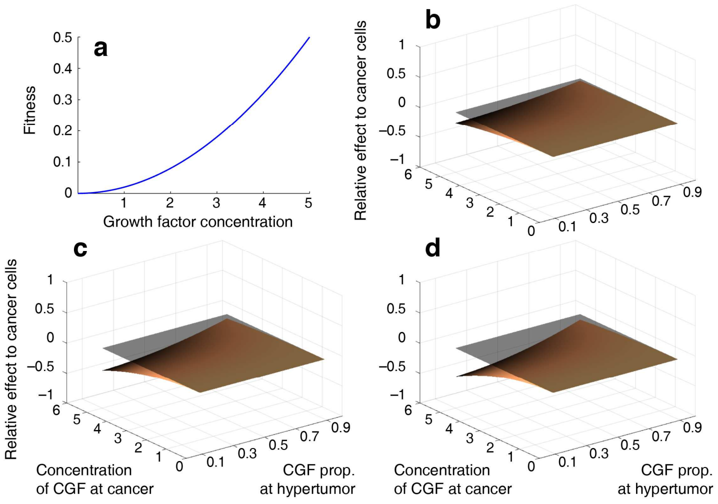

Figure S8. The effect of the anticancer drugs on the progression of the hypertumor at the hypertumor–cancer interface (ϕ) when the fitness function is convex-shaped.

(a) The fitness function in this model is $F(t) = 0.02t^2$ where t is the CGF concentration.

(b) Concentration of CGF at the cancer cells (*y*-axis) varied from 0.1 to 5. The CGF proportion at hypertumor (*x*-axis) indicates the reduced $g(3)/g(1)$ or $g(4)/g(2)$ of the mathematical model. It was assumed that the CGF concentration of cancer cells ($g(1)$, $g(2)$) is higher than that of hypertumor cells ($g(3)$, $g(4)$). Hypertumor progression at the hypertumor–cancer interface is promoted when the anticancer drug is applied if ϕ is negative (shown in *z*-axis). The anticancer drug in this model reduces the CGF by 20%. The transparent gray flat surface indicates $\phi = 0$.

(c) Same as (b) when the anticancer drug reduces the CGF by 50%.

(d) Same as (b) when the anticancer drug reduces the CGF by 80%. The fluctuation of ϕ becomes pronounced as the anticancer effect increases.

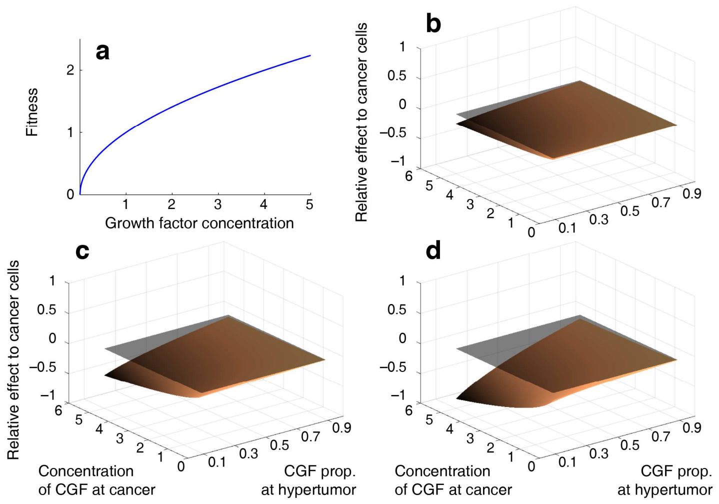

Figure S9. The effect of the anticancer drugs on the progression of the hypertumor at the hypertumor–cancer interface (ϕ) when the fitness function is concave-shaped without an upper bound.

(a) The fitness function in this model is $F(t) = \sqrt{t}$.

(b–d) Panels represent the same information in the panels of Figure S8.

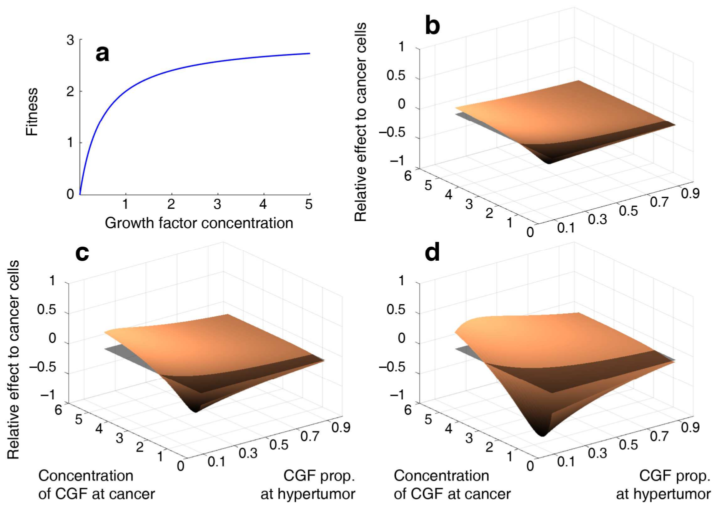

Figure S10. The effect of the anticancer drugs on the progression of the hypertumor at the hypertumor–cancer interface (ϕ) when the fitness function is concave-shaped with an upper bound.

(a) The fitness function in this model is $F(t) = 6t/(2t + 1)$.

(b–d) Panels represent the same information in the panels of Figure S8.

References

1. Martin A Nowak. *Evolutionary dynamics: exploring the equations of life*. (Harvard university press, 2006).
2. Garrido, F. & Aptsiauri, N. Cancer immune escape: MHC expression in primary tumours versus metastases. *Immunology* **158**, 255–266 (2019).
